# Development and validation of a nomogram for predicting unfavorable treatment outcomes in patients with pulmonary tuberculosis and diabetes mellitus

**DOI:** 10.3389/fmed.2026.1722736

**Published:** 2026-01-26

**Authors:** Manman Liu, Tuantuan Li, Haiqing Liu, Fangfang Song, Lili Zhou, Wei Zhang

**Affiliations:** No. 2 People’s Hospital of Fuyang City, Fuyang Infectious Disease Clinical College of Anhui Medical University, Fuyang, Anhui, China

**Keywords:** diabetes mellitus, glucose-to-lymphocyte ratio, nomogram, prognostic model, pulmonary tuberculosis, risk prediction, tuberculosis-diabetes comorbidity

## Abstract

**Objective:**

To develop and validate a clinical prediction model estimating individualized risk of unfavorable treatment outcomes in patients with pulmonary tuberculosis and diabetes mellitus (PTB-DM).

**Methods:**

This retrospective study enrolled 110 inpatients with PTB-DM, categorized into favorable (*n* = 55) and unfavorable (*n* = 55) outcome groups. The Least Absolute Shrinkage and Selection Operator (LASSO) regression was used to select the most relevant predictors from clinical and laboratory data. A multivariate logistic regression model was built based on these predictors to construct a nomogram. The model’s performance was evaluated by its discrimination (Area Under the Curve, AUC), calibration (Hosmer-Lemeshow test and calibration curve), and clinical utility (Decision curve analysis). Internal validation was performed using bootstrap resampling (1,000 repetitions).

**Results:**

Four variables were selected by LASSO regression for model construction: Age, Body Mass Index (BMI), pulmonary cavity, and the Glucose-to-Lymphocyte Ratio (GLR). The multivariate model confirmed these as independent risk factors. The nomogram demonstrated excellent discrimination, with an AUC of 0.885 (95% CI: 0.826–0.944) and a bootstrap-corrected AUC of 0.858. Good calibration was indicated by a non-significant Hosmer-Lemeshow test (*P* = 0.856). Decision curve analysis confirmed the model’s clinical net benefit across a wide range of risk thresholds.

**Conclusion:**

We developed and internally validated a nomogram that accurately predicts the risk of unfavorable outcomes in PTB-DM patients by integrating four readily available clinical parameters. This tool shows robust performance and holds promise for aiding clinicians in identifying high-risk individuals for personalized management strategies.

## 1 Introduction

Tuberculosis (TB) and diabetes mellitus (DM) are two leading global public health challenges, with their epidemics increasingly converging to form a significant co-occurrence burden (1, 2). According to the latest World Health Organization (WHO) report, an estimated 10.7 million new TB cases (95% uncertainty interval: 9.9–11.5 million) and 1.23 million TB-related deaths (95% uncertainty interval: 1.13–1.33 million) were recorded globally in 2024. China remains one of the countries with a high TB burden, accounting for an estimated 696,000 incident TB cases in 2024, which represents approximately 6.5% of the global total (3). Concurrently, the global prevalence of DM is rising at an alarming rate. Data from the International Diabetes Federation (IDF) shows that around 537 million adults worldwide were living with DM in 2021, and this number is projected to increase to 783 million by 2045 (4). Notably, China has the largest DM patient population globally, with an adult DM prevalence of 11.2%, translating to approximately 141 million affected individuals (5). The convergence of these two epidemics results in a substantial population with pulmonary tuberculosis and diabetes mellitus (PTB-DM) comorbidity, posing a significant challenge to global TB control. A systematic review estimated that diabetes mellitus accounts for approximately 15% of pulmonary tuberculosis cases worldwide, with the prevalence of diabetes among TB patients varying from 1.9 to 45% across different regions, and being particularly high in countries like China and India (6). The incidence rates of PTB-DM among Chinese individuals increased from 19.3 to 24.1% (7). This bidirectional relationship not only increases the risk of developing TB but also adversely affects TB treatment outcomes.

Patients with PTB-DM present a unique clinical profile. They often exhibit more severe radiological manifestations, such as a higher prevalence of pulmonary cavities, and experience delayed sputum culture conversion during anti-tuberculosis therapy (8, 9). A key factor contributing to these unfavorable outcomes is the complex interplay between hyperglycemia and the host immune response. Chronic hyperglycemia can lead to dysfunctional innate and adaptive immunity. Specifically, it impairs critical anti-mycobacterial effector functions, such as neutrophil chemotaxis, macrophage phagolysosomal fusion, and antigen presentation, while also promoting a dysregulated cytokine environment (e.g., altered TNF-α, IL-1β, and IFN-γ levels) that is suboptimal for containing Mycobacterium tuberculosis infection (10–12). This dysregulated inflammatory state may be reflected in various hematological parameters.

In recent years, there has been growing interest in using routinely available and cost-effective laboratory indices to assess inflammation and prognosis in various diseases. Indices such as the neutrophil-to-lymphocyte ratio (NLR), systemic immune-inflammation index (SII), and glucose-to-lymphocyte ratio (GLR) have shown promise as biomarkers for disease severity and prognosis in cancers, cardiovascular diseases, and infections (13–15). In the context of TB, some studies have suggested that these inflammatory indices might differ between TB patients with and without DM and could be associated with treatment outcomes (16, 17). Given the dual pathology of metabolic dysregulation (hyperglycemia) and immune impairment (often reflected by lymphopenia) in PTB-DM, we hypothesized that the Glucose-to-Lymphocyte Ratio (GLR), a composite index capturing both axes, might be a particularly relevant prognostic marker worthy of investigation alongside established clinical factors. However, a comprehensive model that integrates these readily available inflammatory-nutritional indices with clinical factors to predict the risk of unfavorable outcomes specifically in PTB-DM patients is still lacking.

Therefore, this study aimed to develop and validate a clinical prediction model for unfavorable treatment outcomes in patients with PTB-DM. We first compared baseline characteristics and a panel of inflammatory-nutritional indices between patients with favorable and unfavorable outcomes. We then employed the Least Absolute Shrinkage and Selection Operator (LASSO) regression to identify the most robust predictors, which were subsequently used to construct a multivariate logistic regression model. Finally, we developed a practical nomogram and rigorously evaluated its discriminative ability, calibration, and clinical utility to facilitate individualized risk stratification in this vulnerable patient population.

## 2 Materials and methods

### 2.1 Study population

This retrospective study was conducted at the Second People’s Hospital of Fuyang City, Anhui Province, China. We employed a case-control design. First, we identified all PTB-DM inpatients with an unfavorable treatment outcome (cases, *n* = 55) who were admitted between January 2024 and December 2024. Subsequently, we performed 1:1 individual matching with PTB-DM patients who had a favorable treatment outcome (controls, *n* = 55) during the same period. Matching was primarily based on admission date (± 3 months) and gender to ensure comparability in temporal trends and basic demographics. This design was chosen to efficiently investigate prognostic factors between these two distinct outcome groups within a limited sample frame from a single center. The treatment outcomes for all patients were assessed after completion of their standard anti-tuberculosis therapy, with data collection performed retrospectively.

The inclusion criteria were as follows: (1) Diagnosis of PTB according to the Chinese National Health Industry Standard (WS 288–2017) (18); (2) Diagnosis of diabetes mellitus based on the American Diabetes Association criteria (19); (3) Availability of complete medical records, including demographic, clinical, imaging, and laboratory data. The exclusion criteria were: (1) Presence of severe psychiatric disorders, malignant tumors, or other severe chronic diseases; (2) Coexistence of other severe infections (e.g., sepsis, severe pneumonia); (3) Incomplete clinical or laboratory data.

All enrolled patients were diagnosed with drug-susceptible pulmonary TB and received standard first-line anti-tuberculosis therapy according to Chinese national guidelines (typically a 2-month intensive phase of isoniazid, rifampin, pyrazinamide, and ethambutol, followed by a 4-month continuation phase of isoniazid and rifampin). Management of diabetes mellitus followed standard clinical practice, including dietary advice, oral hypoglycemic agents, and/or insulin therapy, with the goal of achieving glycemic control.

### 2.2 Sample size determination

Given the case-control design of our study, sample size considerations followed guidelines for prediction model development in matched studies. A widely recommended rule-of-thumb is to have at least 10–20 events (unfavorable outcomes in our case) per candidate predictor variable to ensure model stability and minimize overfitting (20). In our final model, we have 4 predictor variables, and our study includes 55 events (cases), which significantly exceeds the minimum requirement of 40–80 events (10–20 events × 4 predictors). This sample size of 110 participants (55 cases, 55 controls) is therefore considered adequate for the development of a preliminary prediction model, as supported by our robust internal validation results.

### 2.3 Definition of treatment outcome

The primary outcome was “unfavorable treatment outcome,” which referred to the outcome of anti-tuberculosis treatment. It was a composite endpoint defined as any of the following occurring by the end of the standard anti-tuberculosis treatment course (typically 6 months for drug-susceptible TB) or during follow-up within one year after treatment initiation: (1) Treatment failure: lack of conversion of sputum culture to negative by the end of the intensive phase or bacteriological reversion during continuation phase; (2) Death: all-cause mortality during TB treatment; (3) Lost to follow-up: interruption of treatment for ≥ 2 consecutive months; (4) Treatment regimen change due to adverse drug reactions that prevented completion of the standard regimen. Favorable outcome was defined as treatment success, including cure (sputum culture negative in the last month of treatment) and treatment completion (finished treatment without proof of cure but without evidence of failure). Outcome classification was based on all available clinical and follow-up data recorded in the hospital and regional tuberculosis surveillance systems.

### 2.4 Data collection and laboratory measurements

Clinical data were retrospectively collected from the hospital’s electronic medical record system, including: (1) Demographic characteristics: gender, age, body mass index (BMI); (2) Clinical symptoms: cough, fever; (3) Radiological feature: pulmonary cavity (assessed by chest CT or X-ray); (4) Comorbidity: hypertension. Laboratory inflammatory and nutritional indices were calculated from routine complete blood count and biochemical test results. The formulas for these indices are provided in Table 1.

**TABLE 1:** Formulas for calculating laboratory inflammatory indices.

Abbreviation	Full name	Formula
SII	Systemic immune-inflammation index	(Neutrophil count × Platelet count)/Lymphocyte count
SIRI	Systemic inflammation response index	(Neutrophil count × Monocyte count)/Lymphocyte count
NLR	Neutrophil-to-lymphocyte ratio	Neutrophil count/Lymphocyte count
PLR	Platelet-to-lymphocyte ratio	Platelet count/Lymphocyte count
HRR	Hemoglobin-to-red blood cell distribution width ratio	Hemoglobin/Red blood cell distribution width
NPAR	Neutrophil percentage-to-albumin ratio	(Neutrophil percentage%)/Albumin
RAR	Red cell distribution width-to-albumin ratio	Red cell distribution width/Albumin
GLR	Glucose-to-lymphocyte ratio	Glucose/Lymphocyte count

All patients underwent overnight fasting (≥ 8 h) before venous blood sample collection. Serum levels of albumin (ALB) and fasting blood glucose (GLU) were measured using a Hitachi 7600 fully automated biochemical analyzer (Hitachi, Japan). Hematological parameters—including neutrophil percentage (NEUT%), neutrophil count (NEUT), lymphocyte count (LYM), monocyte count (MONO), platelet count (PLT), hemoglobin (HGB), and red blood cell distribution width (RDW)—were analyzed using a Sysmex XE-2100 automated hematology analyzer (Sysmex, Japan).

### 2.5 Statistical analysis

Data statistical analysis and visualization were performed using SPSS 26.0 (IBM Corp., Armonk, NY, United States) and R software (version 4.4.2; R Foundation for Statistical Computing, Vienna, Austria). The normality of continuous variables was assessed using the Shapiro-Wilk test. Normally distributed variables are presented as Mean ± Standard Deviation (SD), non-normally distributed variables as Median (1st Quartile, 3rd Quartile) [M (Q1, Q3)], and categorical variables as frequency (percentage) [n (%)]. Group comparisons were performed using the independent samples *t*-test (for normally distributed continuous variables), the Mann-Whitney U test (for non-normally distributed continuous variables), or the Chi-square test (for categorical variables), as appropriate.

To select predictive variables and avoid overfitting, the Least Absolute Shrinkage and Selection Operator (LASSO) regression method was employed, with the random seed set to 1,234 for reproducibility. The optimal penalty parameter λ (lambda.1se) was identified via 10-fold cross-validation, and variables with non-zero coefficients were retained for subsequent modeling.

Based on the variables selected by LASSO regression, univariate and multivariate logistic regression analyses were conducted to identify independent factors associated with unfavorable outcomes, calculating odds ratios (ORs) and their 95% confidence intervals (CIs). A nomogram prediction model was developed based on the final multivariate model.

The performance of the nomogram was evaluated as follows: discrimination was assessed using the area under the receiver operating characteristic curve (AUC); calibration was evaluated using the Hosmer-Lemeshow goodness-of-fit test and calibration curves; and clinical utility was assessed using Decision curve analysis (DCA) and clinical impact curves. Internal validation was performed using the bootstrap method (1,000 resamples, seed = 123) to obtain a corrected estimate of the model’s AUC. All statistical tests were two-sided, and a *P* < 0.05 was considered statistically significant.

Key R packages utilized included: glmnet (for LASSO regression), rms (for nomogram construction, calibration curves, and DCA), pROC (for ROC analysis), and rmda (for Decision curve analysis).

## 3 Results

### 3.1 Comparison of baseline characteristics between the two groups

The baseline characteristics of all patients are presented in Table 2. Compared with the favorable outcome group (*n* = 55), the unfavorable outcome group (*n* = 55) had significantly higher age (*P* = 0.001) and BMI (*P* < 0.001). The prevalence of pulmonary cavity (*P* < 0.001) and hypertension (*P* = 0.041) was significantly higher in the unfavorable outcome group. Regarding inflammatory and metabolic indicators, the unfavorable outcome group showed significantly elevated NEUT% (*P* < 0.001), NEUT (*P* = 0.005), GLU (*P* < 0.001), SII (*P* = 0.021), NLR (*P* = 0.010), and NPAR (*P* = 0.009), while RDW was significantly lower (*P* = 0.005). Additionally, GLR was significantly higher in the unfavorable outcome group (*P* < 0.001). No statistically significant differences were observed between the two groups in terms of gender, cough, fever, HGB, ALB, HRR, LYM, MONO, PLT, SIRI, PLR, or RAR (all *P* > 0.05).

**TABLE 2:** Comparative analysis of various characteristics of the two groups.

Variables	Total (*n* = 110)	Favorable outcome group (*n* = 55)	Unfavorable outcome group (*n* = 55)	Statistic	*P*
Age, M (Q1, Q3)	55 (45, 64)	50 (36, 61)	58 (52, 67)	*Z* = −3.266	0.001
Gender, n(%)				χ^2^ = 0.183	0.669
Female	30 (27.27)	16 (29.09)	14 (25.45)		
Male	80 (72.73)	39 (70.91)	41 (74.55)		
BMI, Mean ± SD	21.43 ± 3.17	20.44 ± 3.01	22.43 ± 3.03	*t* = −3.459	< 0.001
Cough, n(%)				χ^2^ = 0.043	0.835
No	33 (30.00)	17 (30.91)	16 (29.09)		
Yes	77 (70.00)	38 (69.09)	39 (70.91)		
Fever, n(%)				χ^2^ = 0.052	0.820
No	85 (77.27)	43 (78.18)	42 (76.36)		
Yes	25 (22.73)	12 (21.82)	13 (23.64)		
Pulmonary cavity, n(%)				χ^2^ = 13.285	< 0.001
No	49 (44.55)	34 (61.82)	15 (27.27)		
Yes	61 (55.45)	21 (38.18)	40 (72.73)		
Hypertension, n(%)				χ^2^ = 4.193	0.041
No	85 (77.27)	47 (85.45)	38 (69.09)		
Yes	25 (22.73)	8 (14.55)	17 (30.91)		
NEUT%, Mean ± SD	64.66 ± 11.52	60.64 ± 10.12	68.68 ± 11.52	*t* = −3.890	< 0.001
HGB, Mean ± SD	121.08 ± 16.57	121.71 ± 16.45	120.45 ± 16.82	*t* = 0.395	0.693
ALB, Mean ± SD	37.76 ± 5.91	38.47 ± 5.91	37.05 ± 5.88	*t* = 1.265	0.209
HRR, Mean ± SD	8.59 ± 1.66	8.43 ± 1.66	8.75 ± 1.67	*t* = −1.019	0.311
NEUT, M (Q1, Q3)	4.21 (2.94, 5.74)	3.58 (2.62, 4.97)	4.52 (3.46, 7.04)	*Z* = −2.819	0.005
LYM, M (Q1, Q3)	1.49 (1.06, 1.90)	1.59 (1.17, 1.87)	1.25 (0.96, 1.94)	*Z* = −1.196	0.232
MONO, M (Q1, Q3)	0.55 (0.36, 0.75)	0.55 (0.41, 0.76)	0.51 (0.35, 0.70)	*Z* = −0.795	0.426
RDW, M (Q1, Q3)	14.10 (13.33, 15.07)	14.40 (13.55, 15.30)	13.70 (13.15, 14.65)	*Z* = −2.790	0.005
PLT, M (Q1, Q3)	245.50 (200.00, 292.75)	247.00 (202.00, 297.50)	244.00 (195.00, 292.50)	*Z* = −0.347	0.729
GLU, M (Q1, Q3)	5.07 (4.54, 8.25)	4.72 (4.44, 5.01)	8.23 (5.11, 10.63)	*Z* = −6.035	< 0.001
SII, M (Q1, Q3)	683.44 (399.14, 1182.72)	557.43 (356.34, 959.17)	804.05 (466.32, 1471.89)	*Z* = −2.308	0.021
SIRI, M (Q1, Q3)	1.50 (0.76, 2.76)	1.32 (0.62, 2.21)	1.58 (0.80, 3.66)	*Z* = −1.638	0.101
NLR, M (Q1, Q3)	2.69 (1.91, 4.31)	2.35 (1.68, 3.67)	3.19 (2.15, 5.18)	*Z* = −2.577	0.010
PLR, M (Q1, Q3)	176.74 (117.32, 250.80)	159.17 (122.34, 228.17)	204.79 (111.99, 279.16)	*Z* = −1.055	0.291
NPAR, M (Q1, Q3)	1.72 (1.38, 2.04)	1.64 (1.29, 1.97)	1.87 (1.52, 2.26)	*Z* = −2.624	0.009
RAR, M (Q1, Q3)	0.38 (0.33, 0.43)	0.37 (0.33, 0.44)	0.38 (0.32, 0.43)	*Z* = −0.389	0.698
GLR, M (Q1, Q3)	3.91 (2.78, 6.09)	3.16 (2.43, 4.01)	5.21 (3.42, 8.23)	*Z* = −5.135	< 0.001

*t*, *t*-test; Z, Mann-Whitney test; χ^2^, Chi-square test; SD, standard deviation; M, Median; Q1, 1st Quartile; Q3, 3st Quartile.

### 3.2 Feature selection via LASSO regression

**FIGURE 1:**
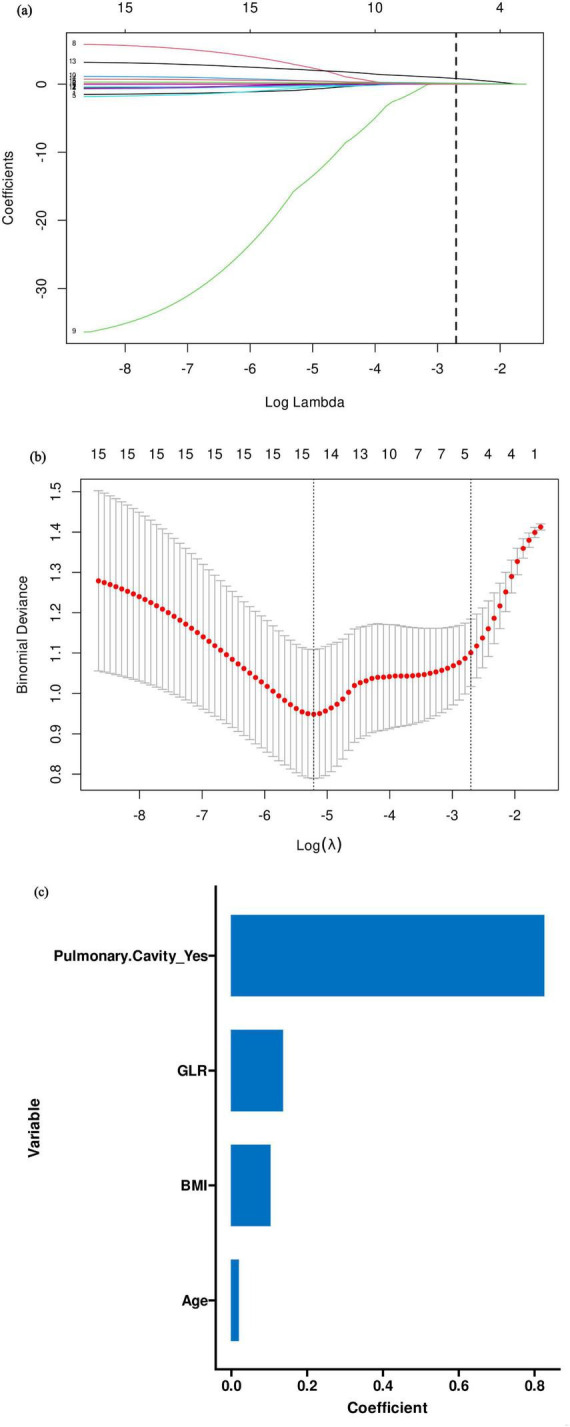
**(a–c)** Clinical feature selection using LASSO regression models.

To identify the most predictive variables, we performed feature selection using the Least Absolute Shrinkage and Selection Operator (LASSO) regression model with a random seed of 1,234. As shown in Figures 1a–c, the optimal model was determined at λ = 0.066935 via 10-fold cross-validation. A total of four variables with non-zero coefficients were selected for subsequent modeling: Age, BMI, pulmonary cavity, and GLR. It is noteworthy that although several other inflammatory indices (e.g., NLR, NEUT%) showed significant differences in univariate analysis, they were not selected by the LASSO algorithm. This suggests that their predictive information may be largely captured by the four retained variables, particularly GLR and pulmonary cavity, highlighting the efficiency of LASSO in handling multicollinearity and selecting the most parsimonious set of predictors.

### 3.3 Univariate and multivariate logistic regression analysis of factors associated with treatment outcomes

To identify independent factors associated with unfavorable outcomes, univariate and multivariate logistic regression analyses were performed. Univariate analysis revealed that Age (OR = 1.056, 95%CI: 1.023–1.089, *P* < 0.001), BMI (OR = 1.250, 95%CI: 1.088–1.435, *P* = 0.002), pulmonary cavity (Yes vs. No: OR = 4.317, 95%CI: 1.930–9.657, *P* < 0.001), and GLR (OR = 1.622, 95%CI: 1.269–2.073, *P* < 0.001) were significantly associated with unfavorable outcomes.

In the multivariate analysis, all four variables remained independent risk factors for unfavorable outcomes: Age (aOR = 1.048, 95%CI: 1.006–1.092, *P* = 0.024), BMI (aOR = 1.332, 95%CI: 1.104–1.607, *P* = 0.003), pulmonary cavity (aOR = 6.958, 95%CI: 2.313–20.932, *P* < 0.001), and GLR (aOR = 1.622, 95%CI: 1.210–2.174, *P* = 0.001). Detailed results are shown in Table 3.

**TABLE 3:** Logistic regression analysis of risk factors associated with treatment outcomes.

Variables	Univariate					Multivariate				
	β	S.E	Wald χ^2^	*P*	OR (95%CI)	β	S.E	Wald χ^2^	*P*	OR (95%CI)
Age	0.054	0.016	3.410	< 0.001	1.056 (1.023 ∼ 1.089)	0.047	0.021	2.251	0.024	1.048 (1.006 ∼ 1.092)
BMI	0.223	0.071	3.153	0.002	1.250 (1.088 ∼ 1.435)	0.286	0.096	2.990	0.003	1.332 (1.104 ∼ 1.607)
**Pulmonary cavity**										
No					1.000 (Reference)					1.000 (Reference)
Yes	1.463	0.411	3.561	< 0.001	4.317 (1.930 ∼ 9.657)	1.940	0.562	3.452	< 0.001	6.958 (2.313 ∼ 20.932)
GLR	0.484	0.125	3.864	< 0.001	1.622 (1.269 ∼ 2.073)	0.484	0.149	3.235	0.001	1.622 (1.210 ∼ 2.174)

CI, confidence interval; OR, odds ratio.

### 3.4 Construction of the nomogram prediction model

**FIGURE 2:**
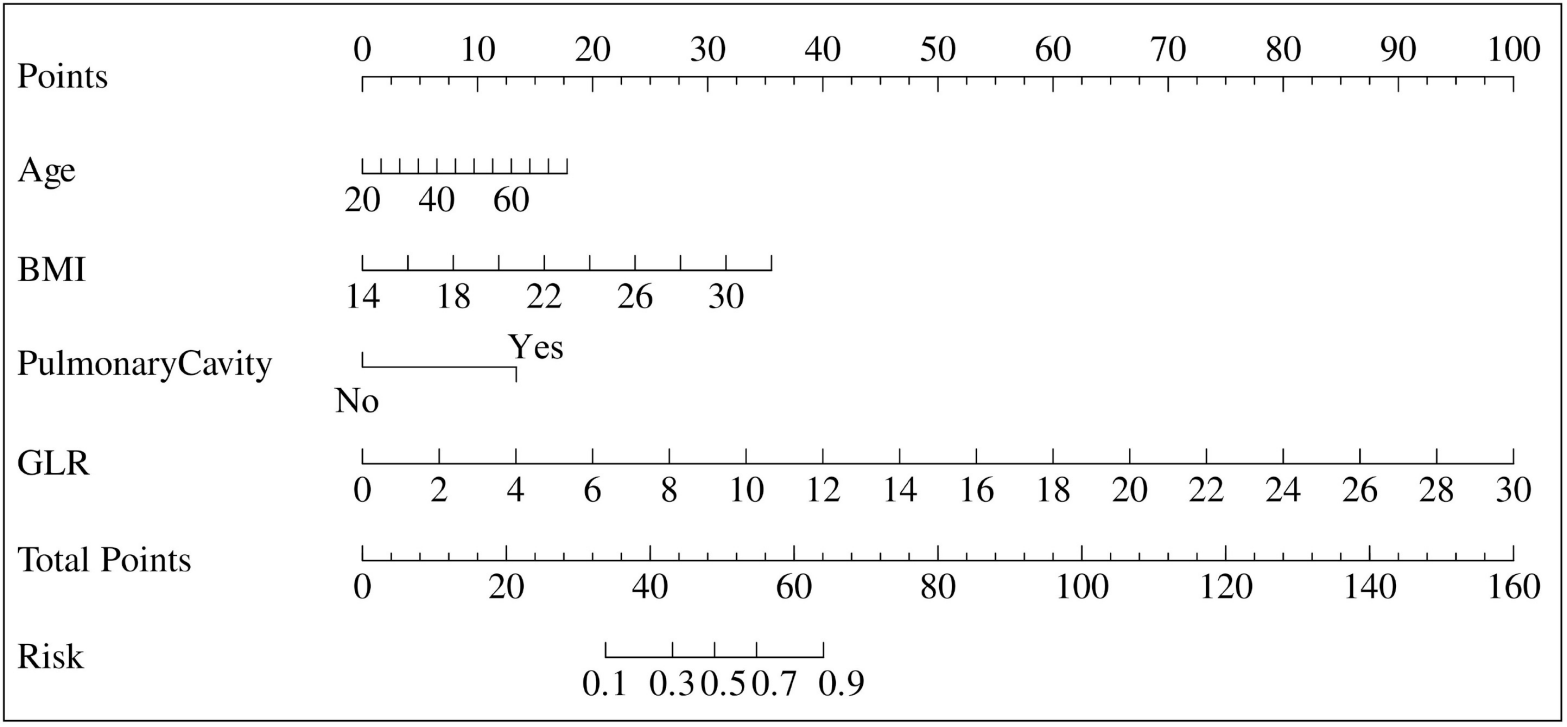
Nomogram model.

Based on the four independent predictors identified by the multivariate logistic regression analysis (Age, BMI, pulmonary cavity, and GLR), a nomogram was developed to individually predict the risk of an unfavorable outcome in PTB-DM patients, as shown in Figure 2. The nomogram is used by first drawing a vertical line upward from each variable’s value to the “Points” axis to obtain its corresponding score. The sum of these four scores yields a “Total Points” value. A vertical line drawn downward from the “Total Points” axis to the “Risk” axis then indicates the individual’s predicted probability of having an unfavorable outcome.

### 3.5 Validation and evaluation of the model

**FIGURE 3:**
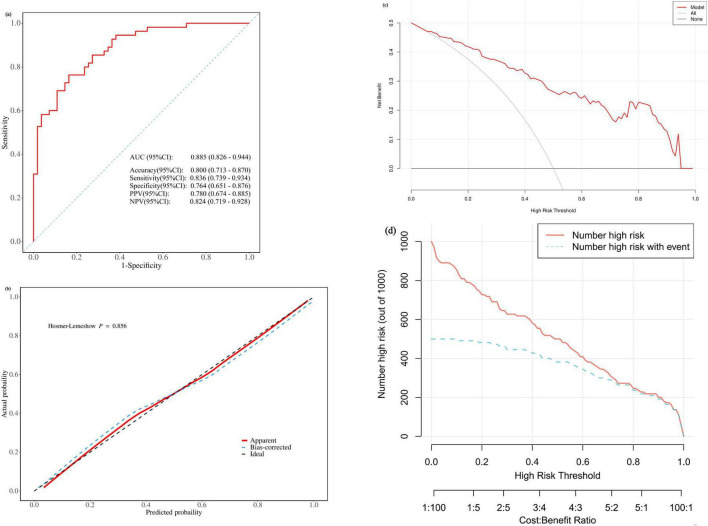
Validation of the nomogram prediction model. **(a)** Receiver operating characteristic (ROC) curve. **(b)** Calibration plot. **(c)** Decision curve analysis (DCA). **(d)** Clinical impact curve. The red solid line (Number high risk) represents the number of patients classified as high risk at each probability threshold by the nomogram. The blue dashed line (Number high risk with event) represents the number of true unfavorable outcomes among those classified as high risk.

The predictive performance of the model was evaluated in terms of discrimination, calibration, and clinical utility. As shown in Figure 3a, the model achieved an area under the receiver operating characteristic curve (AUC) of 0.885 (95% CI: 0.826–0.944) for predicting unfavorable outcomes, with an accuracy of 0.800 (95% CI: 0.713–0.870), sensitivity of 0.836 (95% CI: 0.739–0.934), and specificity of 0.764 (95% CI: 0.651–0.876). Furthermore, internal validation via bootstrap resampling (1000 repetitions, seed = 123) yielded a corrected AUC of 0.858, indicating robust discriminative ability.

The calibration of the model was assessed using the Hosmer-Lemeshow goodness-of-fit test, which yielded a χ^2^ value of 4.015 and a *P*-value of 0.856 (Figure 3b), suggesting no significant deviation between predicted and observed probabilities. The calibration curve in Figure 3b also demonstrated close alignment between the bootstrap-corrected predictions and the ideal line.

Decision curve analysis (DCA) revealed (Figure 3c) that across a wide range of threshold probabilities (approximately 0.1–0.9), the use of this prediction model provided higher net benefit than the “treat-all” or “treat-none” strategies, supporting its clinical utility. The clinical impact curve (Figure 3d) further confirmed that across different risk thresholds, the number of true events among those classified as high risk followed a consistent trend with the total number of high-risk individuals, underscoring the model’s value for clinical risk stratification.

## 4 Discussion

The convergence of the tuberculosis (TB) and diabetes mellitus (DM) epidemics presents a significant clinical challenge, particularly in high-burden countries like China (21). Patients with PTB-DM face a substantially higher risk of unfavorable treatment outcomes, yet a reliable tool for individualized risk stratification has been lacking. In this retrospective study, we developed and validated a clinical prediction model for unfavorable outcomes in PTB-DM patients. By integrating both clinical features and a novel inflammatory-nutritional index, we identified four independent predictors—Age, BMI, pulmonary cavity, and GLR—and incorporated them into a user-friendly nomogram. The model demonstrated robust discrimination, good calibration, and promising clinical utility. The following discussion will interpret our key findings in the context of existing literature, explore their clinical implications, acknowledge the study’s limitations, and suggest directions for future research.

Our findings align with and extend previous research on risk factors for poor TB outcomes. The association between older age and unfavorable outcomes is well-documented, likely due to immunosenescence and a higher prevalence of comorbidities (22). Similarly, the strong independent association of pulmonary cavity with unfavorable outcomes is a classic feature of severe TB, often linked to high bacillary load and delayed sputum conversion, a correlation that is particularly pronounced in PTB-DM cohorts (23). Our study identified higher BMI as an independent risk factor for unfavorable outcomes, which appears to contrast with the conventional association of low BMI and malnutrition with poor TB prognosis. This paradox underscores the distinct pathophysiology of the PTB-DM comorbidity. Crucially, the mean BMI in our cohort was within the normal range (21.4 kg/m^2^), indicating that this association is relevant even in the absence of clinical obesity. This finding suggests that variations in body composition within the “normal” BMI range may have significant biological implications in PTB-DM. BMI does not differentiate between muscle mass and fat mass. In patients with diabetes, a higher BMI, even within the normal spectrum, may reflect a greater proportion of adiposity relative to lean mass. Adipose tissue, particularly visceral fat, is a potent endocrine organ secreting pro-inflammatory adipokines (e.g., leptin, TNF-α, IL-6) (24). In the context of pre-existing diabetic dysmetabolism, this adiposity-associated low-grade inflammation can be exacerbated, leading to a state of “meta-inflammation” that dysregulates both innate and adaptive immune responses crucial for controlling M. tuberculosis infection (25). Therefore, in our PTB-DM patients, the detrimental immunomodulatory effects driven by excess adiposity-related inflammation might have offset the potential protective benefits of better nutritional reserves, leading to the observed association. Future studies incorporating body composition analysis (e.g., waist-hip ratio, bioelectrical impedance) are warranted to dissect this relationship further. The identification of the Glucose-to-Lymphocyte Ratio (GLR) as a powerful predictor is a notable finding. While hyperglycemia is known to impair lymphocyte function (26), and lymphopenia has been associated with worse TB outcomes (27), the composite index GLR synergistically captures both metabolic dysregulation and immune incompetence. This may explain its superior performance over using glucose or lymphocyte count alone in our model. It is noteworthy that although univariate analysis showed significant associations for certain inflammatory indices such as NLR, they were not retained in the final multivariate model. This suggests that their predictive information may be encompassed by the stronger contributors, namely GLR and pulmonary cavity. Future studies should prospectively validate GLR in larger, multi-center cohorts to confirm its generalizability. Furthermore, the optimal clinical cut-off value for GLR needs to be determined in larger cohorts to translate this continuous variable into a actionable clinical tool.

The discriminative performance of our nomogram, with an AUC of 0.885 (0.858 after bootstrap correction), compares favorably with existing prognostic models for tuberculosis. For instance, Lu et al. developed a model to predict poor outcomes in the intensive phase of general pulmonary tuberculosis treatment, reporting an AUC of 0.815 (28). More importantly, in a direct comparison within the TB-DM population, You et al. constructed a risk score for poor outcomes among TB-DM patients in Eastern China, achieving an AUC of 0.850 (29). Our model, which integrates the novel inflammatory-metabolic index (GLR) with key clinical features (age, BMI, and pulmonary cavity), demonstrated a higher AUC (0.885). While differences in study populations and predictor sets preclude a definitive head-to-head comparison, the superior discriminative ability of our nomogram suggests that the inclusion of GLR-a marker capturing both dysglycemia and immune status-may provide added predictive value beyond traditional demographic and clinical factors alone. This underscores the potential of combining metabolic and inflammatory biomarkers for risk stratification in this comorbid condition.

The primary application of our research lies in its potential to facilitate personalized patient management. The constructed nomogram provides a practical tool for clinicians to quantitatively estimate the risk of an unfavorable outcome for an individual PTB-DM patient at the time of diagnosis. A high predicted risk could justify more intensive monitoring, longer treatment duration, more aggressive glycemic control, or even a different drug regimen, as suggested by some guidelines for severe TB cases (30). Furthermore, the GLR is calculated from routine, low-cost laboratory tests, making this model highly feasible for implementation in resource-limited settings where the PTB-DM burden is highest. By enabling early identification of high-risk patients, this model could help allocate limited healthcare resources more efficiently and ultimately improve treatment success rates in this vulnerable population. From an implementation perspective, the nomogram could be integrated into hospital Electronic Medical Record (EMR) systems as a clinical decision support module. Upon entering a new PTB-DM patient’s Age, BMI, pulmonary cavity status (from radiology report), and routine lab results (glucose and lymphocyte count), the EMR could automatically calculate the GLR and the total nomogram score, displaying the predicted risk category (e.g., low, intermediate, high) to alert the treating physician. This would facilitate point-of-care risk assessment without requiring additional calculations.

## 5 Limitations

Despite its promising results, our study has several limitations. First, its retrospective and single-center design inherently introduces potential selection bias and limits the generalizability of our findings. Importantly, our matched case-control design means that the model estimates the relative odds of an unfavorable outcome rather than the absolute risk. Translation of the nomogram score into an absolute risk estimate for clinical use necessitates external validation in a prospective cohort. Second, the sample size, while meeting the minimum requirement for model development, remains modest. Our sample size calculation relied on an anticipated Cox-Snell R^2^ of 0.2, which is inherently uncertain. Although we employed techniques like LASSO regression and bootstrap validation to mitigate overfitting, the limited sample size may have constrained our statistical power to identify other potential predictors with weaker effects and could affect the stability of the coefficient estimates. A larger cohort would be valuable to refine the model and enhance its generalizability. Third, our model does not include microbiological data, such as drug susceptibility testing results, which are critical determinants of treatment outcome. The inclusion of only drug-susceptible TB patients was a control measure, but in real-world settings, drug resistance is a major confounder that future iterations of the model must incorporate. Four, we used a single fasting blood glucose measurement at admission to calculate GLR. This reflects acute glycemia but not long-term glycemic control (e.g., HbA1c). We lacked data on diabetes duration and detailed glycemic management history. Therefore, we cannot rule out that the difference in GLR between groups is influenced by the chronicity and severity of diabetes itself, which could be a confounder. Future models should incorporate HbA1c and diabetes duration. Finally, the definition of unfavorable outcome, while clinically relevant, grouped together several distinct endpoints (e.g., treatment failure, death). The predictors for each specific endpoint might differ.

Based on the limitations discussed, future research should prioritize the external validation of our nomogram in diverse populations. Prospective studies are needed to ascertain if intervention strategies guided by the nomogram actually lead to improved patient outcomes. Incorporating microbiological data (e.g., drug resistance), more detailed glycemic control metrics (e.g., HbA1c trajectory during treatment), and other omics data could further enhance the model’s accuracy and clinical relevance.

## 6 Conclusion

In conclusion, we have developed and internally validated a novel nomogram that integrates Age, BMI, pulmonary cavity, and GLR to predict the risk of unfavorable outcomes in patients with PTB-DM at treatment initiation. This model demonstrates excellent predictive performance and has the potential to serve as a practical tool for clinicians to identify high-risk individuals, thereby facilitating timely interventions and personalized treatment strategies to improve patient prognosis.

## Data Availability

The original contributions presented in this study are included in this article/Supplementary material, further inquiries can be directed to the corresponding author.

## References

[1] Jeong D Mok J Jeon D Kang H Kim H Kim H Prevalence and associated factors of diabetes mellitus among patients with tuberculosis in South Korea from 2011 to 2018: a nationwide cohort study. *BMJ Open.* (2023) 13:e069642. 10.1136/bmjopen-2022-069642 36889835 PMC10008237

[2] Gurumurthy M Gopalan N Patel L Davis A Srinivasalu V Rajaram S Treatment outcomes in people with diabetes and multidrug-resistant tuberculosis (MDR TB) enrolled in the STREAM clinical trial. *PLoS Glob Public Health.* (2025) 5:e0004259. 10.1371/journal.pgph.0004259 40168299 PMC11960897

[3] World Health Organization. *Global Tuberculosis Report 2025.* Geneva: World Health Organization (2025).

[4] Alshahrani A Vasudevan R Aldahish A Alghazwani Y Shorog E Manusri N Analyzing foot care practices and diabetes management: a cross-sectional KAP study in a tertiary care hospital. *Front Endocrinol.* (2025) 16:1547366. 10.3389/fendo.2025.1547366 40510479 PMC12158677

[5] Liu X Zhang L Chen W. Trends in economic burden of type 2 diabetes in China: based on longitudinal claim data. *Front Public Health.* (2023) 11:1062903. 10.3389/fpubh.2023.1062903 37143967 PMC10151735

[6] Noubiap J Nansseu J Nyaga U Nkeck J Endomba F Kaze A Global prevalence of diabetes in active tuberculosis: a systematic review and meta-analysis of data from 2.3 million patients with tuberculosis. *Lancet Glob Health.* (2019) 7:e448–60. 10.1016/S2214-109X(18)30487-X 30819531

[7] Shi H Yuan Y Li X Li Y Fan L Yang X. Analysis of the influencing factors and clinical related characteristics of pulmonary tuberculosis in patients with type 2 diabetes mellitus. *World J Diabetes.* (2024) 15:196–208. 10.4239/wjd.v15.i2.196 38464376 PMC10921156

[8] Xu R Zhang Y Li Z He M Lu H Liu G Breathomics for diagnosing tuberculosis in diabetes mellitus patients. *Front Mol Biosci.* (2024) 11:1436135. 10.3389/fmolb.2024.1436135 39193220 PMC11347294

[9] Chen W Ding Q Zhang S Tong Z Ren F Hu C Nutritional status in patients with active pulmonary tuberculosis and new nutritional risk screening model for active tuberculosis: a national, multicenter, cross-sectional study in China. *J Thorac Dis.* (2023) 15:2779–99. 10.21037/jtd-23-623 37324100 PMC10267931

[10] Kinsella R Kimmey J Smirnov A Woodson R Gaggioli M Chavez S Autophagy prevents early proinflammatory responses and neutrophil recruitment during Mycobacterium tuberculosis infection without affecting pathogen burden in macrophages. *PLoS Biol.* (2023) 21:e3002159. 10.1371/journal.pbio.3002159 37319285 PMC10306192

[11] Nafiz T Sankar P Mishra L Rousseau R Saqib M Subbian S Differential requirement of formyl peptide receptor 1 in macrophages and neutrophils in the host defense against Mycobacterium tuberculosis Infection. *Sci Rep.* (2024) 14:23595. 10.1038/s41598-024-71180-1 39384825 PMC11464745

[12] Ahor H Schulte R Adankwah E Harelimana J Minadzi D Acheampong I Monocyte pathology in human tuberculosis is due to plasma milieu changes and aberrant STAT signalling. *Immunology.* (2023) 170:154–66. 10.1111/imm.13659 37219921

[13] Shao L Yu B Lyu Y Fan S Gu C Wang H. The clinical value of novel inflammatory biomarkers for predicting Mycoplasma pneumoniae infection in children. *J Clin Lab Anal.* (2025) 39:e25150. 10.1002/jcla.25150 39800911 PMC11821716

[14] Zhang W Xiang C Liu B Hou F Zheng Z Chen Z The value of systemic immune inflammation index, white blood cell to platelet ratio, and homocysteine in predicting the instability of small saccular intracranial aneurysms. *Sci Rep.* (2024) 14:24312. 10.1038/s41598-024-74870-y 39414876 PMC11484959

[15] Liu L Zhang B Li Y Huang W Niu Y Jia Q Preoperative glucose-to-lymphocyte ratio predicts survival in cancer. *Front Endocrinol.* (2024) 15:1284152. 10.3389/fendo.2024.1284152 38501103 PMC10946689

[16] Zahid M Afaq S Shafique K Qazi F Ashfaq U Asim M Effect of glycemic control on tuberculosis treatment outcomes among patients with tuberculosis and diabetes mellitus: a systematic review and meta-analysis. *Trop Med Int Health.* (2025) 30:749–62. 10.1111/tmi.14140 40551384 PMC12318439

[17] Zhao L Gao F Zheng C Sun X. The impact of optimal glycemic control on tuberculosis treatment outcomes in patients with diabetes mellitus: systematic review and meta-analysis. *JMIR Public Health Surveill.* (2024) 10:e53948. 10.2196/53948 38564244 PMC11022131

[18] National Health Commission of the People’s Republic of China. Diagnostic criteria for pulmonary tuberculosis WS 288—2017. *J Tuberc Lung Dis.* (2024) 5:376–8. 10.19983/j.issn.2096-8493.2024022

[19] American Diabetes Association Professional Practice Committee. 2. Diagnosis and classification of diabetes: standards of care in diabetes-2024. *Diabetes Care.* (2024) 47:S20–42. 10.2337/dc24-S002 38078589 PMC10725812

[20] Peduzzi P Concato J Kemper E Holford T Feinstein AR. A simulation study of the number of events per variable in logistic regression analysis. *J Clin Epidemiol.* (1996) 49:1373–9. 10.1016/s0895-4356(96)00236-3 8970487

[21] Song W Shao Y Liu J Tao N Liu Y Zhang Q Primary drug resistance among tuberculosis patients with diabetes mellitus: a retrospective study among 7223 cases in China. *Infect Drug Resist.* (2019) 12:2397–407. 10.2147/IDR.S217044 31447568 PMC6684854

[22] Nahid P Jarlsberg L Rudoy I de Jong B Unger A Kawamura L Factors associated with mortality in patients with drug-susceptible pulmonary tuberculosis. *BMC Infect Dis.* (2011) 11:1. 10.1186/1471-2334-11-1 21199579 PMC3022714

[23] Wang W Wang X Chen S Li J Cheng Q Zhang Y Prevalence and clinical profile of comorbidity among newly diagnosed pulmonary tuberculosis patients: a multi-center observational study in eastern China. *Front Med.* (2025) 12:1446835. 10.3389/fmed.2025.1446835 39935798 PMC11810726

[24] Ellulu M Patimah I Khaza’ai H Rahmat A Abed Y. Obesity and inflammation: the linking mechanism and the complications. *Arch Med Sci.* (2017) 13:851–63. 10.5114/aoms.2016.58928 28721154 PMC5507106

[25] Ssekamatte P Sitenda D Nabatanzi R Nkurunungi G Nakibuule M Kibirige D Metabolic dysfunction impairs Mycobacterium tuberculosis-specific cytokine and chemokine responses in latent tuberculosis and type 2 diabetes mellitus. *Sci Rep.* (2025) 15:30474. 10.1038/s41598-025-16385-8 40830413 PMC12365027

[26] Wu G Ke H Tong Z Yang J Yang J Shen Z. Association between glucose-to-lymphocyte ratio and mortality in patients with heart failure from the MIMIC-IV database: a retrospective cohort study. *Sci Rep.* (2025) 15:21131. 10.1038/s41598-025-08349-9 40595315 PMC12219531

[27] Li F Chen D Zeng Q Du Y. Possible mechanisms of Lymphopenia in severe tuberculosis. *Microorganisms.* (2023) 11:2640. 10.3390/microorganisms11112640 38004652 PMC10672989

[28] Lu B Shi Y Wang M Jin C Liu C Pan X Development of a clinical prediction model for poor treatment outcomes in the intensive phase in patients with initial treatment of pulmonary tuberculosis. *Front Med.* (2025) 12:1472295. 10.3389/fmed.2025.1472295 40206468 PMC11978639

[29] You N Pan H Zeng Y Lu P Zhu L Lu W A risk score for prediction of poor treatment outcomes among tuberculosis patients with diagnosed diabetes mellitus from eastern China. *Sci Rep.* (2021) 11:11219. 10.1038/s41598-021-90664-y 34045573 PMC8160203

[30] Nahid P Mase S Migliori G Sotgiu G Bothamley G Brozek J Treatment of drug-resistant tuberculosis. An official ATS/CDC/ERS/IDSA clinical practice guideline. *Am J Respir Crit Care Med.* (2019) 200:e93–142. 10.1164/rccm.201909-1874ST 31729908 PMC6857485

